# COVID-19 mRNA Vaccine Effectiveness against Elderly Frail People

**DOI:** 10.3390/medicina59020202

**Published:** 2023-01-19

**Authors:** Jannis Kountouras, Maria Tzitiridou-Chatzopoulou, Apostolis Papaefthymiou, Dimitrios Chatzopoulos, Michael Doulberis

**Affiliations:** 1 Second Medical Clinic, School of Medicine, Ippokration Hospital, Aristotle University of Thessaloniki, 54642 Thessaloniki, Greece; 2 Midwifery Department, School of Healthcare Sciences, University of West Macedonia, Koila, 50100 Kozani, Greece; 3 Department of Gastroenterology, University Hospital of Larisa, 41110 Larisa, Greece; 4 Division of Gastroenterology and Hepatology, Medical University Department, Kantonsspital Aarau, 5001 Aarau, Switzerland

**Keywords:** COVID-19 mRNA vaccine, effectiveness, immunological effects, old age, frailty

## Abstract

The frail, elderly population is often characterized by poor immunogenicity post COVID-19 mRNA vaccination. “Inflame-ageing” and “immune-senescence” are pathogenetic mechanisms that might explain this phenomenon. Complex interplay with cytokines and microbiota is also implicated in this inflammatory cascade. The abovementioned population, although very important from immunologic perspective, has barely been included in the mRNA vaccination clinical trials.

## Foreword

Recent evidence indicates that frailty can independently predict the reduced antibody response after COVID-19 mRNA vaccines in elderly people [1]. More specifically, post-vaccination reaction is weaker and even uncommon in this age group when compared with young people; and serious adverse events are more common [2]. Although reduced vaccine efficacy in the setting of frailty is well defined, the immunological background of frailty is partially described, thereby demanding further research. 

In this regard, future studies have to consider, beyond other parameters, the potential involvement of “inflamm-ageing”, “immune-senescence”, and alterations of gut microbiota (i.e., gut dysbiosis) in inducing an altered response to COVID-19 mRNA vaccination in frail elderly people.

“Inflamm-ageing” and “immune-senescence” are risk factors for severe COVID-19 in elderly persons. The sterile inflammatory process, or “inflamm-ageing”, is involved in the pathophysiology of several age-associated pathologies. These pathologies include atherosclerosis, rheumatoid arthritis, diabetes mellitus, Alzheimer’s disease and/or senescence itself. Moreover, when SARS-CoV-2 infection co-exists with the aforementioned age-associated pathologies, COVID-19 appears to be more severe and/or life-threatening [3,4]. 

“Inflamm-ageing” is a systemic multifactorial condition, characterized by complex interactions owing to an excess of molecular mediators, as exemplified by the nuclear factor (NF)-κB interactome. NF-κB appears to influence the age-linked deficiencies of innate and adaptive immune systems, related to “inflamm-ageing” (mediated principally by the innate immune arm) and diminishing adaptive immunity (via inherent deficiencies of lymphocytes in signaling and proliferation), respectively [5].

Specifically, “inflamm-ageing”, which is related to the increased cellular production and secretion of pro-inflammatory cytokines into circulation (e.g., interleukin (IL)-6, IL-1, and tumor necrosis factor (TNF)-α), in combination with the diminished concentration of anti-inflammatory cytokines (e.g., IL-10 and transforming growth factor beta), is linked with gut dysbiosis [6]. Markers of “inflamm-ageing” include the long-term stimulation of innate immunity and fluctuating concentrations of the above-mentioned pro-inflammatory and anti-inflammatory cytokines, respectively. Regarding microbiota changes, studies have recently revealed prominent gut dysbiosis among COVID-19 patients [7], detected even in patients with COVID-19 naïve to antibiotic therapy, which is characterized by the enhancement of opportunistic pathogens and the depletion of beneficial commensals. Fecal microbiota alterations are connected to higher fecal concentrations of SARS-CoV-2 and a severe form of COVID-19 [8]. The prolonged gut dysbiosis in COVID-19 is linked with fecal SARS-CoV-2 shedding and disease severity [8]. 

The perpetual crosstalk among the innate and adaptive immune systems and the resultant continuing “inflamm-ageing” are responsible for the frailty that accompanies the aging population of society, predisposing them to susceptibility to infections, including SARS-CoV-2. Frailty, which essentially is of a multidimensional and multisystemic nature, results in a noticeable susceptibility to a cluster of adverse health-linked events. Such events include injuries, infections, disability, hospitalization, and mortality [9]. In this regard, the mentioned gut dysbiosis is responsible for severe forms of COVID-19 [8]. Severe forms of COVID-19 present an increased concentration in the mentioned pro-inflammatory cytokines associated with end-organ damage and lethality. Older COVID-19 patients are exposed to risk of all-cause fatality [10]. Moreover, the deceased patients infected by COVID-19 displayed alterations in lung microbiota (i.e., lung dysbiosis), particularly in age ≥65 years and/or with comorbidities such as metabolic syndrome-related severe disorders. In particular, fatal COVID-19 is related to complex mixed bacterial and fungal infections in the lungs [11]. Recent meta-transcriptome sequencing of bronchoalveolar lavage fluid shows that the microbiota in SARS-CoV-2-infected patients is dominated by pathogens of the oral cavity and upper respiratory tract [12]. In this respect, hospitalized old COVID-19 patients exhibit increased respiratory microbiota dysbiosis than healthy arms. A possibility of gut–respiratory microbiome crosstalk or migration could exist. The disturbance among gastrointestinal and lung microbiota may further deteriorate the host immune responses in episodes of SARS-CoV-2 infection, resulting in an unrestrained inflammatory process [13]. Lung dysbiosis might play a significant role in the pathogenetic mechanisms of severe forms of COVID-19 and could enhance the risk of over-infections connected with COVID-19. A main mechanism by which lung microbiota dysbiosis during the COVID-19 affects the degree of the disease appears to be the alterations in the innate and adaptive immune reactions, including pro-inflammatory cytokines and B- and T-lymphocytes.

Moreover, although elderly people have been included in the priority lists for the COVID-19 vaccinations, frail elderly patients were possibly excluded from COVID-19 vaccination-related studies. Likewise, “inflamm-ageing” and “immune-senescence” are linked with a reduced immune reaction against the vaccination or preceding infections and this reduced response could be worse among frail elderly people [14]; adaptive immune system declines by the age, as confirmed by decreased responsiveness to vaccination [15]. This recognized impaired response to vaccinations among frail elderly people has motivated clinicians to assess for frailty before deciding whether to introduce mRNA vaccination or not. In a recent study, it was reported that 23 frail elderly patients succumbed soon after receiving mRNA vaccination [16]. Comparable data also indicate that aged people (≥80 years), as well as populations with multimorbidity and certain concomitant health problems are at augmented risk of COVID-19 related hospitalization and mortality following the initial vaccine booster of BNT162b2 or mRNA-1273 vaccine; these data recognize risk factors (e.g., ageing and immunosuppression), and emphasize the significantly raised risk postulated by multimorbidity [17]. Additional data also indicate that, following the second dose of vaccines, humoral response is significantly declined, particularly among males, populations ≥65 years old, and immunosuppressed populations. Peak antibody responses are observed in the first month and then decline to sub-quarter levels at ten weeks post vaccination. Likewise, six months after receiving the second dose of vaccine, the humoral response is significantly reduced [18]. Additional data also indicate that antibody responses after a booster dose tend to decline rapidly in older populations (>60 years) after an initial peak: a sub-five-fold drop in peak antibody titer occurs in 16 weeks [19]. Therefore, the longevity of COVID-19 vaccination-related immunogenicity is rather lacking [20]. Furthermore, in populations aged from 18 to 74 years, COVID 19-related vaccines could be linked to the enhanced occurrence of myocardial infarction and pulmonary embolism [21].

Interestingly, recent in vitro studies indicate that vaccine-related SARS-CoV-2 spike protein considerably reduces the DNA damage repairing proteins, which are essential for efficient V(D)J recombination in adaptive immunity. In this way, vaccines weaken the reactions of adaptive immune system and suggest a probable adverse event of spike protein-based vaccines [20]; mutations of such repair proteins appear to trigger oncogenic process [22]. Moreover, recent published data revealed an excess of risk of serious adverse effects related to mRNA-based COVID-19 vaccines and underlined the necessity of harm–benefit analyses, in order to stratify risk [23]. More specifically, patients ≥65 years old have been linked with a higher rate of post-vaccination hospitalizations, death, and life-threatening outcomes, than populations age 18–64 years (relative risk estimates among 1.49 99% CI [1.44–1.55] and 8.61 99% CI [8.02–9.23]). Moreover, when compared with influenza vaccines, COVID-19 vaccines yielded raised relative risks for allergic reactions, arrhythmia, general cardiovascular events, coagulation, hemorrhages, constitutional, gastrointestinal, ocular, sexual organs reactions, and, in particular, thromboembolic events [24]. In a nationwide mass vaccination background, including and elderly participants, the BNT162b2 vaccine was connected with an excess risk of myocarditis (11.0 events per 100,000 persons) and of other severe adverse outcomes, including myocardial infarction, pericarditis, arrhythmia, deep vein thrombosis, pulmonary embolism, intracranial hemorrhage, and thrombocytopenia [25]. 

Interestingly, recent data on autopsies of patients 46–75 years old documented the development of myocarditis as a fatal complication following mRNA-based anti-SARS-CoV-2 vaccination up to 20 days prior to their death. Importantly, none of the dead patients had COVID-19 before vaccination [26].

Considering myocardial infarction, its incidence, after COVID-19 vaccination, increased significantly among patients aged >85 years when compared with younger arms (1400 and 28 per 100,000 person years, respectively) [27]. COVID-19 post-vaccination myocardial infarction has occurred very rarely in children, rarely in women aged 35–54 years, infrequently in men and women aged 55–84 years, and frequently in those aged ≥85 years [27]. Likewise, acute kidney injury occurred most frequently in elderly patients after the COVID-19 vaccines [27].

In frail elderly people there is a recognized connection between DNA repair defects, genomic instability, oxidative stress, and age-related disorders including malignancies. In this regard, beyond reduced response to COVID-19 vaccinations, both “inflamm-ageing” and “immune-senescence” induce an increased susceptibility of the elderly to malignancies, infectious diseases, autoimmune or cardiovascular disorders, and dementia. Therefore, the latter data suggest that vaccine-related SARS-CoV-2 spike protein may inhibit adaptive immunity and emphasize the possible adverse events of full-length spike-based vaccination.

The aforementioned considerations might raise concerns regarding the safety of initiating COVID-19 vaccinations, including the mRNA vaccination, without adequate evidence from long-term large-scale clinical studies in this special frail elderly population. Since some authors may claim that there is no serious risk for excluding frail elderly people from vaccination on safety grounds, further related research is warranted to elucidate this crucial topic in depth.

## Data Availability

Not applicable.
